# AGO2 Negatively Regulates Type I Interferon Signaling Pathway by Competition Binding IRF3 with CBP/p300

**DOI:** 10.3389/fcimb.2017.00195

**Published:** 2017-05-22

**Authors:** Shengyu Wang, Xin Sun, Chenyang Yi, Dan Zhang, Xian Lin, Xiaomei Sun, Huanchun Chen, Meilin Jin

**Affiliations:** ^1^State Key Laboratory of Agricultural Microbiology, Huazhong Agricultural UniversityWuhan, China; ^2^Laboratory of Animal Virology, College of Veterinary Medicine, Huazhong Agricultural UniversityWuhan, China; ^3^Key Laboratory of Development of Veterinary Diagnostic Products, Ministry of Agriculture, College of Veterinary Medicine, Huazhong Agricultural UniversityWuhan, China; ^4^The Cooperative Innovation Center for Sustainable Pig ProductionWuhan, China

**Keywords:** AGO2, negative regulation, type I interferon, IRF3, CBP/p300

## Abstract

Viral infection triggers a series of signaling cascades and host innate immune responses, including interferon (IFN) production, which depends on coordinated activity of multiple transcription factors. IFN regulatory factor 3 (IRF3) and transcriptional coactivator CREB binding protein (CBP) and/or p300 are core factors that participate in transcriptional complex formation in the nucleus. In general, cells balance the production of IFNs through suppressive and stimulative mechanisms, but viral infections can disrupt such equilibrium. This study determined that H5N1 viral infection reduced the distribution of human argonaute 2 (AGO2) in A549 cell nucleus. AGO2 did not block phosphorylation, nuclear translocation, and DNA binding ability of IRF3 but inhibited its association with CBP. Therefore, this newly revealed mechanism shows that cellular response leads to transfer of AGO2 from cell nucleus and promotes IFN-β expression to increase host survival during viral infection.

## Introduction

Viral infection results in activation of innate and multistep immune responses. These responses require recognition of viral infections, activation of multiple signal transduction cascades, and transcription of antiviral genes (Akira et al., 2006; Katze et al., 2008; Goubau et al., 2013). Interferon beta (IFN-β) is one of the most important proteins and is a critical component of innate immune response; most cells of the body secrete this type of molecule (Akira et al., 2006; Ivashkiv and Donlin, 2014). During cell infection, viruses are recognized by intracellular pattern recognition receptors (PRRs), which include Toll-like receptors, retinoic acid-inducible gene I (RIG-I)-like receptors and nucleotide-binding oligomerization domain like receptors. PRRs activate innate immune signaling pathways and increase activities of transcription factors (Akira et al., 2006; Gilliet et al., 2008; Chiang et al., 2014; Wu and Chen, 2014). These transcription factors include IFN regulatory factor 3 (IRF3), nuclear factor kappa B, activator protein 1, and the coactivator CREB binding protein (CBP) and/or p300. Together, these transcription factors can form complexes that bind to regulatory domains of the IFN-β promoter and induce transcription of IFN-β (Yoneyama et al., 1998; Gough et al., 2012; Ivashkiv and Donlin, 2014).

IFN-β is tightly regulated in many ways, and its aberrant secretion can trigger diseases, such as multiple sclerosis and systemic lupus erythematosus. Regulation of IRF3, a key protein in the IFN-β pathway, includes important positive and negative mechanisms (Gonzalez-Navajas et al., 2012; Ivashkiv and Donlin, 2014; Ysebrant de Lendonck et al., 2014). The positive mechanisms comprise increased expression and activation of IRF3, and the negative ones involves its decreased expression and inactivation (Servant et al., 2002; Ysebrant de Lendonck et al., 2014; Wang et al., 2015; Meng et al., 2016). IFN-β expression is also regulated by formation of a transcriptional complex containing IRF3 and CBP/p300 (Yoneyama et al., 1998; Servant et al., 2002).

IRF3 is the most important factor in regulation of viral induced IFN-β activation. In general, IRF3 is found in the cytoplasm in latent form (Ysebrant de Lendonck et al., 2014). When viruses expose pathogen-associated molecular patterns and are recognized by PRRs, IRF3 is activated to promote antivirus reaction (Akira et al., 2006; Chiang et al., 2014; Ivashkiv and Donlin, 2014). Activation of IRF3 includes C-terminal region phosphorylation, dimerization, and translocation to the nucleus (Servant et al., 2002; Ysebrant de Lendonck et al., 2014).

Studies extensively investigated the mechanisms underlying phosphorylation-induced activation of IRF3 (Lin et al., 1998, 1999; Yoneyama et al., 1998; Servant et al., 2001, 2003; Mori et al., 2004; Chen et al., 2008; Clement et al., 2008; Takahasi et al., 2010). IRF3 is phosphorylated on multiple phosphorylation acceptor (phospho-acceptor) sites. Previous studies showed that phosphorylation of C-terminal phospho-accepter clusters (Ser385-Ser386 and Ser396-Ser398-Ser402-Thr404-Ser405) plays important role in activation of IRF3 (Lin et al., 1998, 1999; Yoneyama et al., 1998; Servant et al., 2003; Mori et al., 2004). Other research also showed that phosphorylation of Ser339 on IRF3 is involved in stabilizing IRF-3, and mutation of Ser339 can abrogate CBP association, and dimerization (Saitoh et al., 2006; Clement et al., 2008). However, Ser339 mutation does not affect gene transactivation as long as Ser396 is available for phosphorylation (Clement et al., 2008). Previously published data indicated that Ser386 and Ser396 play important roles in IRF3 activity, and Ser339 is not integrant under physiological conditions.

Transcriptional coactivators CBP and p300 are critical regulators of metazoan gene expression (Lin et al., 2001; Suhara et al., 2002). Transcriptional coactivators associate with different DNA-bound transcription factors through small conserved domains (Lin et al., 2001). A compactly folded 46-residue domain in CBP serves as the IRF3 binding domain (IBiD), and interaction occurs at the C terminus domain of IRF3 (Lin et al., 2001). Activated IRF3 interacts with coactivator CBP/p300 and initiates transcription of IFN-β (Yoneyama et al., 1998; Suhara et al., 2002; Reily et al., 2006; Ysebrant de Lendonck et al., 2014). Phosphorylation at Ser386 and Ser396 plays significant roles in IRF3 activation and its interaction with CBP (Chen et al., 2008; Takahasi et al., 2010). Following phosphorylation and dimerization, IRF3 translocates to the nucleus and recruits CBP and/or p300 for full activation (Yoneyama et al., 1998; Suhara et al., 2002; Ysebrant de Lendonck et al., 2014; Song et al., 2016). Then, this complex binds to PRD I/III in the IFN-β promoter for transcription activation and IFN-β production (Weaver et al., 1998).

Human argonaute 2 (AGO2) is a multifunctional protein that interact with many molecules (Hock and Meister, 2008; Martinez and Gregory, 2013). Recent studies on the structure of AGO2 have revealed the four core domains including N domain, PAZ domain, MID domain, and PIWI domain (Kuhn and Joshua-Tor, 2013; Ye et al., 2015). AGO2 is a key regulator and activator that performs specific siRNA/miRNA-dependent and -independent functions. This protein also interacts with Dicer, TAR RNA binfing protein (TRBP) and PACT, which are required to construct the RNA-induced silencing complex-loading complex and to process precursor miRNAs into mature miRNAs (Chendrimada et al., 2005; Martinez and Gregory, 2013; Ye et al., 2015). AGO2 also inhibits translation initiation via interaction with eukaryotic initiation factor 6 (eIF6) and prevents recruitment of the translation initiation factor eIF4-E by binding to 7-methylguanosine cap (Chendrimada et al., 2007; Kiriakidou et al., 2007). AGO2 binds to AU element of 3'-untranslated region of TNF (i.e., TNF-alpha) mRNA and upregulates translation under serum starvation (Vasudevan and Steitz, 2007). AGO2 is also normally located on euchromatin instead of heterochromatin, and once combined with CTCF/CP190, it can inhibit related target gene expression through CTCF/CP190-dependent Fab-8 insulation (Moshkovich et al., 2011). Studies on cellular functions of AGO2 should thus be continued.

Previous studies showed that the proteins associated with RNA interference (RNAi) pathway may influence viral replication. For instance, PACT enhances the function of RIG-I, and TRBP influences PKR, these phenomena can affect innate immunity and viral proliferation (Cosentino et al., 1995; Kok et al., 2011; Kim et al., 2014). Dicer functions as effector and sensor by cleaving viral double-stranded RNAs and by activating downstream signaling or effector systems that generate relatively more robust and amplified antiviral responses (Ahmad and Hur, 2015). Dicer also reduces influenza viral replication, which is siRNA/miRNA independent (Matskevich and Moelling, 2007). This study further investigated AGO2 function during viral infection. This study reveals that AGO2 participates in negative regulation of IFN-β signaling pathway in a novel manner, inhibiting formation of transcription initiation complex and consequent IFN-β production.

## Materials and methods

### Cell culture and transfection

Madin-Darby canine kidney (MDCK) cells were obtained from the American Type Culture Collection (ATCC) and maintained in Dulbecco's minimal essential medium (DMEM; Invitrogen, Carlsbad, CA, USA) with 10% fetal bovine serum (FBS; PAN biotech, Auckland, New Zealand) and 5% CO_2_ at 37°C. Human embryonic kidney (HEK293T) cells were maintained in RPMI 1640 medium (HyClone, China) with 10% FBS, and human type II alveolar epithelial (A549) cells were propagated in Ham's F12K medium (F-12, HyClone, China) with 10% FBS and 5% CO_2_ at 37°C.

For the transient overexpression of specific proteins, cells were transfected using Lipfection2000 (Invitrogen) and analyzed at 36 h or 48 h post transfection. For gene silencing, AGO2 siRNA (siAGO2) and control siRNA (siNC) were obtained from Transheep (Transheep, Shanghai, China). Cells were transfected with siRNAs using Lipfection2000 following the manufacturer's protocol at a final concentration of 100 nM.

### Antibodies and plasmids

The antibodies used in this study were sourced from the following: mouse monoclonal Flag antibody was from Sigma (MO, USA); mouse monoclonal HA antibody was from MBL (Japan); rabbit polyclonal HA antibody and rabbit polyclonal IRF3 antibody were from ABclonal Biotechnology (China); rabbit polyclonal Ser396 phosphorylated IRF3 antibody was from Merck Millipore (Germany); rabbit polyclonal Ser386 phosphorylated IRF3 antibody, mouse monoclonal AGO2 antibody [2E12-1C9], rabbit monoclonal AGO2 antibody [EPR10410] and rabbit monoclonal LaminA/C antibody [EPR4100] were from Abcam (Cambridge, UK); CBP (D6C5) Rabbit mAb and Myc-Tag (9B11) Mouse mAb were from Cell Signaling Technology (USA); mouse anti-β-actin polyclonal antibody, mouse anti-β-tubulin polyclonal antibody and mouse anti-GAPDH polyclonal antibody were from BioPM (Wuhan, China); fluorescein isothiocyanate (FITC)-labeled goat anti-mouse secondary antibody, Cy3-labeled goat anti-rabbit secondary antibody and horseradish peroxidase (HRP) conjugated secondary antibodies were from PKR (China).

Flag-RIG-I, Flag-VISA, Flag-TBK-1 and HA-IRF3 expression plasmids and a luciferase (Luc) reporter plasmid for the IFN-β promoter (IFN-β-Luc) were kindly provided by Zhengfan Jiang (Peking University, China) (You et al., 2009). A Renilla control plasmid (pGL4.75 hRluc/CMV, where CMV is a cytomegalovirus) (Promega) was used to control for the cell number and transfection efficiency. HA-AGO2 and Flag-AGO2 was amplified from HEK293T cDNA and inserted into pCAGGS-HA expression vector and p3 × FLAG-CMV-14 expression vector. Flag-IRF3 was cloned from HA-IRF3 and inserted into p3 × FLAG-CMV-14 expression vector. Flag-RIG-I-N, Flag-IRF3-5D, FLAG-IRF3(1–197), Flag-IRF3(198–427), Flag-IRF3-5D(198–427), and Myc-IBiD were generated as previously described and were cloned into pCAGGS-HA expression vector, p3 × FLAG-CMV-14 expression vector or pCMV-C-Myc expression vector (Lin et al., 1998, 1999, 2001; Yoneyama et al., 2005). The four domain of AGO2 were amplified from HA-AGO2 and inserted into pCAGGS-HA expression vector (HA-AGO2-N, HA-AGO2-PAZ, HA-AGO2-MID, and HA-AGO2-PIWI) as previously described (Kuhn and Joshua-Tor, 2013). The MID deleted AGO2 were cloned from HA-AGO2 and inserted into pCAGGS-HA expression vector (HA-AGO2-MIDdel). All plasmids constructs were verified by DNA sequencing.

### TCID_50_ assays

Cell supernatants containing the virus were serially diluted 10-fold with DMEM and applied in quadruplicate to 2 × 10^4^ MDCK cells/well in a 96-well plate. On the fifth day post infection, the viral titer was determined by observing the cytopathogenic effect and was confirmed by hemagglutination. The TCID_50_ was determined based on the Reed–Muench method as described previously (Ramakrishnan, 2016).

### Influenza virus preparation and infection of cells

Wild-type H5N1 virus A/Hubei/hangmei01/2006 (H5N1) and Sendai virus (SeV) were grown in 10-day-old fertilized eggs. The working stocks were stored at −80°C as live viruses or after inactivation by formaldehyde treatment. The viral titer of H5N1 was measured using the Reed–Muench method (Ramakrishnan, 2016). H5N1 virus experiments were performed in Biosafety Level 3 facilities at the State Key Laboratory of Agricultural Microbiology, College of Veterinary Medicine, Huazhong Agricultural University, China.

For H5N1 infection, A549 cells were washed three times in F-12 to remove FBS and then incubated with influenza virus diluted in F-12 for 1 h at 37°C. After 1 h, the cells were washed and maintained in F-12 with 1% FBS for the indicated times. For IFN-β expression, HEK293T cells and A549 cells were stimulated with SeV and analyzed at 3, 6, 12, or 24 h later.

### Luciferase reporter assay

HEK293T cells in 12-well plates were transfected with 0.5 μg of IFN-β-Luc and 0.01 μg of pGL4.75 hRluc/CMV with 0.5, 1 μg, and/or 2 μg of AGO2 expressing plasmids; 24 h post transfection, cells were stimulated with SeV for 12 h and then lysed in 200 μl of passive lysis buffer (PLB; Promega). HEK293T cells in 12-well plates were transfected with siAGO2 and, 36 h later, the cells were transfected with 0.5 μg of IFN-β-Luc and 0.01 μg of pGL4.75 hRluc/CMV; after another 24 h post transfection, cells were stimulated with SeV for 12 h and then lysed in 200 μl of passive lysis buffer. HEK293T cells in 12-well plates were transfected with 0.5 μg of IFN-β-Luc; 0.01 μg of pGL4.75 hRluc/CMV; 0.5 μg of RIG-I, RIG-I-N, VISA, TBK1, IRF3, or IRF3-5D; and 2 μg of the AGO2 expressing plasmids using 6 μl of Lipofectamine 2000. The cells were incubated for 24 h and then lysed in 200 μl of passive lysis buffer. Luciferase and Renilla activities were assessed using a Dual-Luciferase Assay Kit (Promega).

### Quantitative RT-PCR

Total cellular RNA was extracted using TRIzol (Life Technologies, USA) and treated with DNase using RQ RNase-free DNase (Promega, China) prior to cDNA production. The cDNA was reverse-transcribed from 1 μg of total RNA using oligo(dT) primers according to the manufacturer's protocol (TaKaRa, China). Quantitative RT-PCR was carried out using SYBR Green Master Mix (Roche) and specific primer sets (Table 1). Amplification reactions were performed under the following conditions: 2 min at 50°C, 10 min at 95°C, 40 cycles for 15 s at 95°C, and 1 min at 60°C. Relative transcript levels were calculated using the ΔΔCt method as specified by the manufacturer.

**Table 1:** **List of primers for different genes**.

**Gene name**	**Forward primer sequence**	**Reverse primer sequence**
GAPDH	GACAACTTTGGTATCGTGGAA	CCAGGAAATGAGCTTGACA
IFN-β	CTCTCCTGTTGTGCTTCTCC	GTCAAAGTTCATCCTGTCCTTG
Mx1	GGTGGTCCCCAGTAATGTGG	CGTCAAGATTCCGATGGTCCT
IFIT1	GCGCTGGGTATGCGATCTC	CAGCCTGCCTTAGGGGAAG
ISG15	TGGACAAATGCGACGAACCTC	TCAGCCGTACCTCGTAGGTG
OAS1	AGCTTCGTACTGAGTTCGCTC	CCAGTCAACTGACCCAGGG
STAT1	CGGCTGAATTTCGGCACCT	CAGTAACGATGAGAGGACCCT
AGO2	GTTTGACGGCAGGAAGAATCT	AGGACACCCACTTGATGGACA
NP	CAGCGTTCAGCCCACTTTCT	GGGTTCGTTGCCTTTTCGTC

*All of the sequence are from 5′–3′*.

### Immunoprecipitation

Cells were lysed in RIPA lysis buffer (CST, USA) containing protease inhibitors (Calbiochem) for 30 min on ice and were then centrifuged at 12,000 rpm for 15 min. The supernatant was incubated with antibody for 1 h at 4°C and the lysate-antibody complexes were incubated with Protein A/G PLUS-Agarose (Santa Cruz Biotechnology, Europe) overnight at 4°C. The precipitated agarose was washed four times with lysis buffer to remove nonspecific binding. The immune complex was eluted with 2 × SDS Loading buffer and boiled, separated on SDS-PAGE, and analyzed by Western analysis. The Bradford assay was used for protein quantification.

### Immunofluorescence and microscopy

To visualize the subcellular localization of IRF3 and AGO2, cells were mock infected or infected with SeV. The cells were then fixed with 4 % paraformaldehyde for 15 min and permeabilized with 0.1% Triton X-100 for 15 min at room temperature. After three washes with phosphate-buffered saline (PBS), the cells were blocked with PBS containing 3% BSA at 37°C for 1 h. The cells were then incubated separately with rabbit polyclonal IRF3 antibody (1:100) and mouse monoclonal AGO2 antibody (1:200) overnight at 4°C. Subsequently, the cells were treated with FITC-labeled goat anti-mouse secondary antibody and Cy3-labeled goat anti-rabbit secondary antibody (1:500) for 1 h and then stained with DAPI (1:1,000, Beyotime, China) for 15 min at room temperature. After the samples were washed with PBS, fluorescent images were acquired with a confocal laser scanning microscope (Carl Zeiss, Göttingen, Germany).

### Subcellular fractionation

For subcellular fractionation, the previously reported protocol was modified and used in this study. A portion of the cells were lysed with RIPA buffer containing protease inhibitor. The samples were frozen and thawed repeatedly and then centrifuged at 12,000 rpm at 4°C for 15 min. The supernatants were kept as whole cell samples. The remaining cells from the dishes were lysed with 125 μl of hypotonic lysis buffer (10 mM Hepes-NaOH (pH 7.9), 10 mM KCl, 1.5 mM MgCl2, 0.5 mM beta-mercaptoethanol) supplemented with protease inhibitor mixture and phosphatase inhibitors, vortexed and then incubated on ice. After 15–20 min, the mixture received an additional 3 μl 10% NP-40, was vortexed, placed on ice for 2 min and centrifuged at 16,000 g at 4°C for 10 min. This supernatant was kept as cytoplasmic extract. To prepare nuclear extracts, nuclear pellets were washed two times with 1 ml of ice-cold PBS and were resuspended in 60 μl of RIPA buffer as nuclear lysis buffer. The samples were centrifuged at 16,000 g at 4°C for 10 min, and the supernatant was kept as nuclear extract. Proteins were quantified by a Bradford assay, and a western blot was conducted as previously described.

### DNA binding assay

The DNA probes labeled with biotin were synthesized by Tsingke BioTech. The sequence of triple repeats of IRF3-recognized and bound ISRE contained biotin-3 × ISRE-F (biotin-TAGTTTCACTTTCCCTAGTTTCACTTTCCCTAGTTTCACTTTCCC) and 3 × ISRE-R (GGGAAAGTGAAACTAGGGAAAGTGAAACTAGGGAAAGTGAAACTA), and the sequence of triple repeats of mutated IRF3-recognized and bound ISRE contained biotin-3 × ISRE-mutant-F (biotin-TAGTTTCA**G**TTTCCCTAGTTTCA**G**TTTCCCTAGTTTCA**G**TTTCCC) and 3 × ISRE-mutant-R (GGGAAA**C**TGAAACTAGGGAAA**C**TGAAACTAGGGAAA**C**TGAAACTA) (mutation in bold and underlined). Single strands of DNA were thermally annealed to form dsDNA prior to pull-down experiments. HEK293T cells were transfected with Flag-IRF3 in the absence or presence of HA-AGO2, and cells were infected with SeV at 24 h post transfection. Twelve hours later, lysates were mixed with biotinylated DNA probes and incubated for 1 h at 4°C. BeaverBeadsTM Streptavidin beads (BEAVER, China) were then added and incubated overnight at 4°C. The beads were washed four times with PBS and were resolved by 2 × SDS loading buffer for western blot.

### Statistical analyses

All of the data are shown as mean ± SD. Statistical analyses were conducted using the Student's *t*-test for two groups. A *p* < 0.05 was considered significant.

## Results

### AGO2 increases H5N1 virus replication

**Figure 1:**
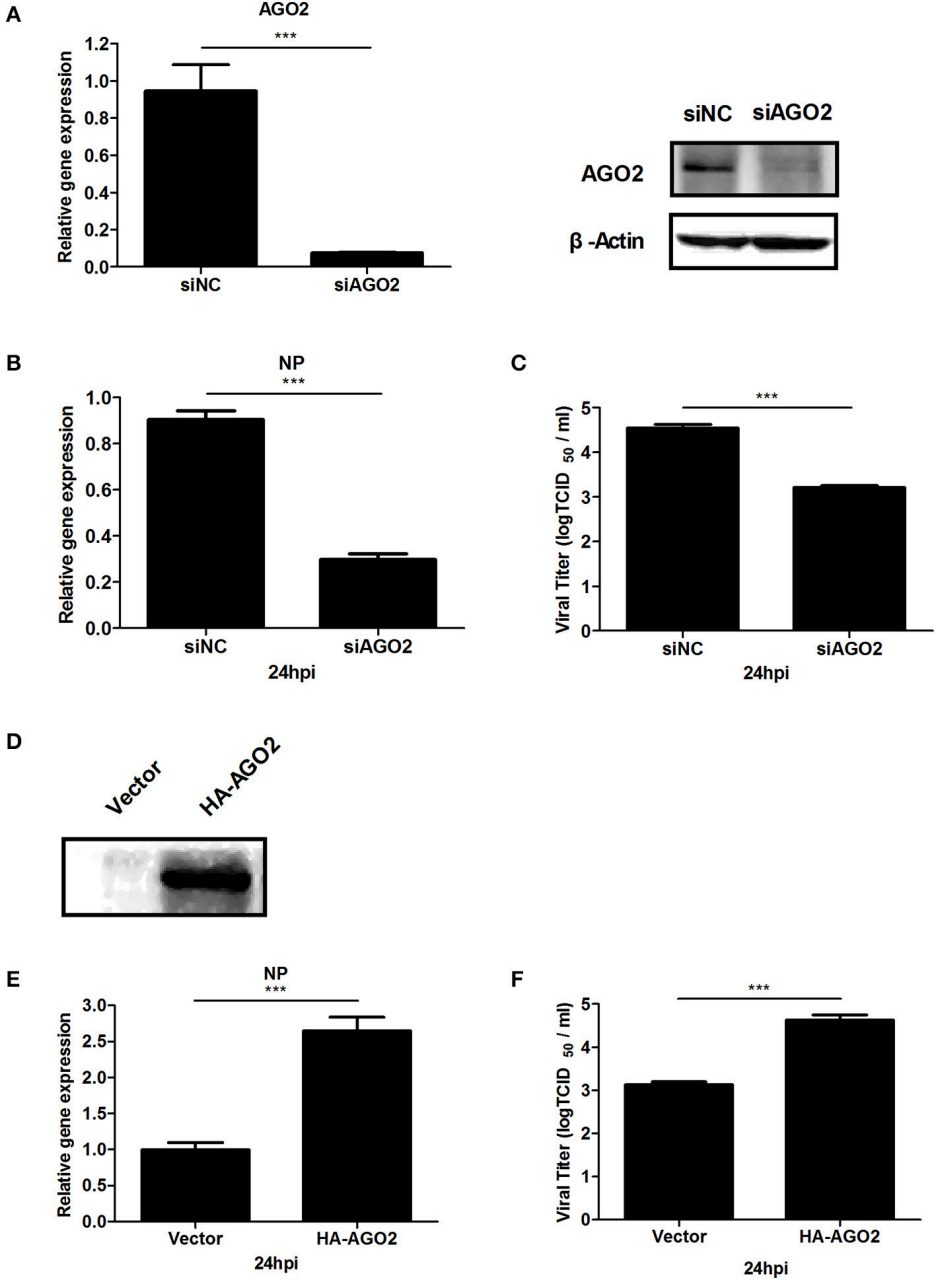
**AGO2 promotes proliferation of H5N1 in A549 cells. (A)** A549 cells were transfected with siNC or siAGO2. After 48 h, the cells were harvested and examined. Quantitative RT-PCR and western blot were used to assess silencing efficiency. Data are presented as means ± SD from three independent experiments. **(B,C)** A549 cells were transfected with siNC or siAGO2. After 48 h, the cells were infected with H5N1. Quantitative RT-PCR for H5N1 NP gene mRNA and TCID_50_ assays for the virus titer of H5N1 were performed to detect multiplication of the virus at 24 h.p.i. Data are presented as means ± SD from three independent experiments. **(D)** A549 cells were transfected with HA-AGO2 and empty vectors, and expressions was detected by western blot. **(E,F)** A549 cells were transfected with HA-AGO2 and empty vectors. After 48 h, the cells were infected with H5N1. Quantitative RT-PCR for H5N1 NP gene mRNA and TCID_50_assays for the virus titer of H5N1 were performed to estimate virus multiplication at 24 h.p.i. Data are presented as means ± SD from three independent experiments. ^***^P < 0.0001, as determined by a *t* test.

**Figure 2:**
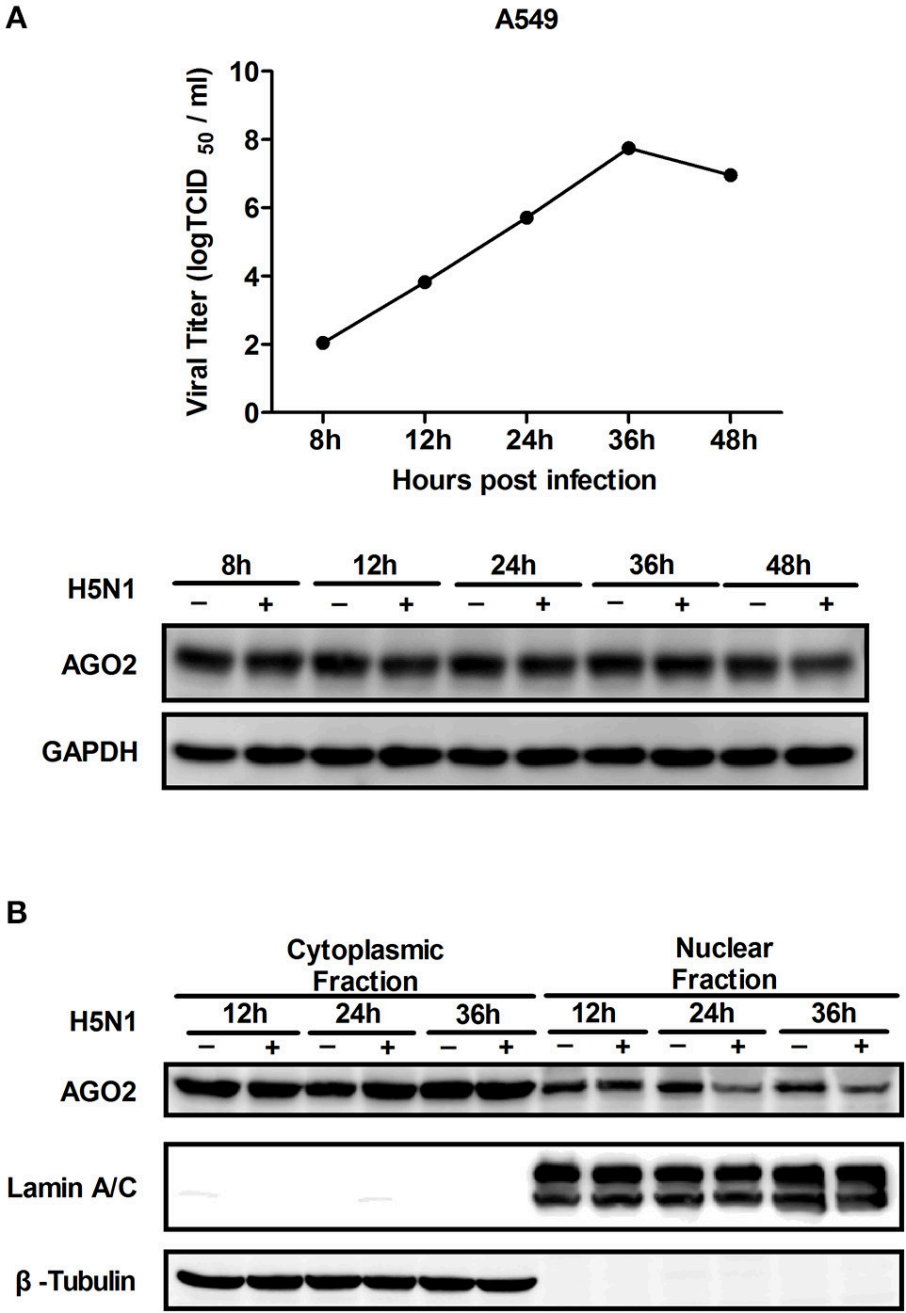
**Expression and distribution of AGO2 in cells. (A)** A549 cells were infected with H5N1. Kinetics of virus infection and protein levels of AGO2 were detected at different time points. **(B)** A549 cells were infected with H5N1. At different time points, cells were harvested, subcellular fractionation was performed, and AGO2 in cells was detected by Western blot.

AGO2 expression was modified, and H5N1 virus titer in A549 cells was detected to investigate whether AGO2, a key protein in the RNAi pathway, also influences virus propagation. First, siRNA was designed against the human AGO2, and siAGO2 knockdown efficiency was confirmed by quantitative RT-PCR and western blot (Figure 1A). Then, A549 cells were transfected with siAGO2 or nonspecific control siRNA (siNC) and infected with H5N1 after 48 h. At 24 h post infection (h.p.i.), viral NP gene mRNA levels and viral titers were evaluated in H5N1 virus infected A549 cells. NP gene mRNA levels were significantly reduced in the siAGO2 group compared with that in control (Figure 1B). Viral titer in siAGO2-treated cells was determined with TCID_50_ assay, and the obtained value was also lower than that of siNC-treated cells at 24 h.p.i. (Figure 1C). These results indicated that AGO2 knockdown inhibited replication of H5N1 virus. The effect of AGO2 overexpression on viral replication was also determined by transfecting A549 cells with HA-AGO2. Efficacy of AGO2 overexpression was determined by western blot (Figure 1D). As determined by TCID_50_ assay, AGO2-overexpressing groups yielded higher NP gene mRNA level and virus titer than the control group (Figures 1E,F). Protein levels of AGO2 in cells, cytoplasm, and cell nucleus were detected to explore the functional role of AGO2 in virus/host interactions. Results showed reduced protein level of AGO2 in cell nucleus during H5N1 infection (Figures 2A,B). These data indicated that AGO2 promoted replication of H5N1 virus, and that its distribution may influence virus/host interactions.

### AGO2 participates in IFN signaling pathway

**Figure 3:**
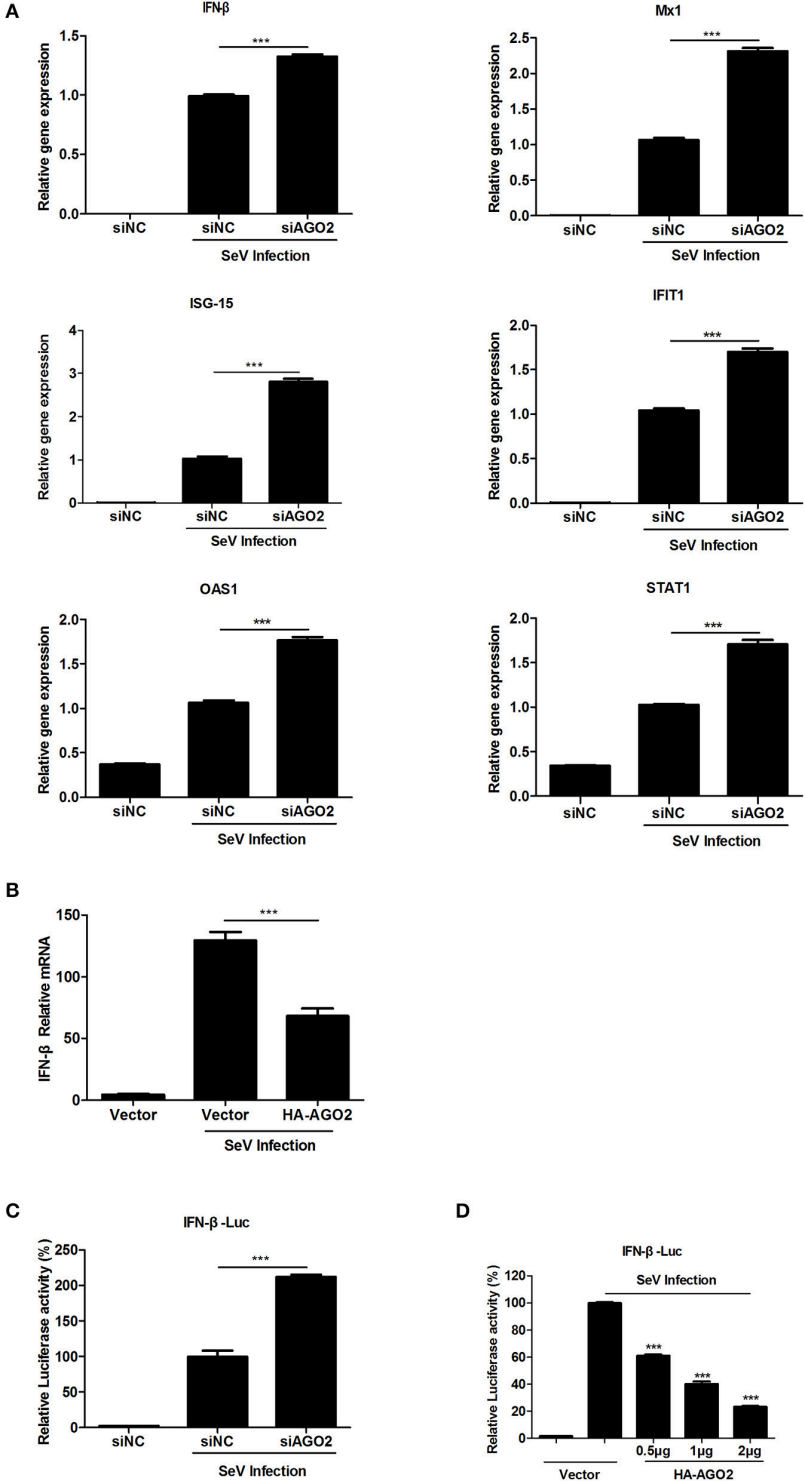
**AGO2 inhibits SeV induced IFN-β activation. (A)** A549 cells were transfected with siNC and siAGO2. After 48 h, the cells were stimulated with SeV, and RNA expression levels of the ISGs and IFN-β were compared. Data are presented as means ± SD from three independent experiments. **(B)** A549 cells were transfected with HA-AGO2 and an empty vector. After 48 h, the cells were stimulated with SeV, and the RNA expression levels of IFN-β were examined. Data are presented as means ± SD from three independent experiments. **(C)** HEK293T cells were cotransfected with siNC/siAGO2, IFN-β-Luc, and pGL4.75 (hRluc/CMV). After 48 h, the cells were stimulated with SeV, and luciferase reporter assay was performed. Data are presented as means ± SD from three independent experiments. **(D)** HEK293T cells were transfected with IFN-β-Luc and pGL4.75 hRluc/CMV together with increasing quantities of plasmids (i.e., 0.5, 1, and 2 μg) encoding for AGO2. At 24 h post transfection, the cells were further infected with SeV or mock infected for 16 h before luciferase assays were performed. Data are presented as the means ± SD from three independent experiments. ^***^P < 0.0001, as determined by a *t* test.

Previous studies showed that RNAi pathway associated proteins, including PACT and TRBP, regulate IFNs (Cosentino et al., 1995; Kok et al., 2011). This study showed that AGO2 enhances multiplication of H5N1 virus and speculated that AGO2 possibly affects IFN signaling pathway. To investigate this hypothesis on AGO2, quantitative RT-PCR was performed, and comparison were made on changes in mRNA expression levels of IFN-stimulated genes (ISGs) and IFN-β between AGO2 and control knocked down A549 cells stimulated with SeV. Data showed that silencing of AGO2 increased the expression levels of endogenous IFN-β and downstream IFIT1 and of ISGs such as Mx1, STAT1, and ISG15 (Figure 3A). Assessment showed significantly reduced endogenous IFN-β level with overexpression of AGO2 in SeV-simulated A549 cells (Figure 3B). Double fluorescence reporting system in HEK293T cell showed the same results, and that AGO2 caused dose-dependent inhibition of IFN-β promoter activity (Figures 3C,D). These results indicated that AGO2 inhibited expressions of IFN-β in both A549 cells and HEK293T cells.

### Targets of inhibitory effect of AGO2 in type I IFN signaling pathway

**Figure 4:**
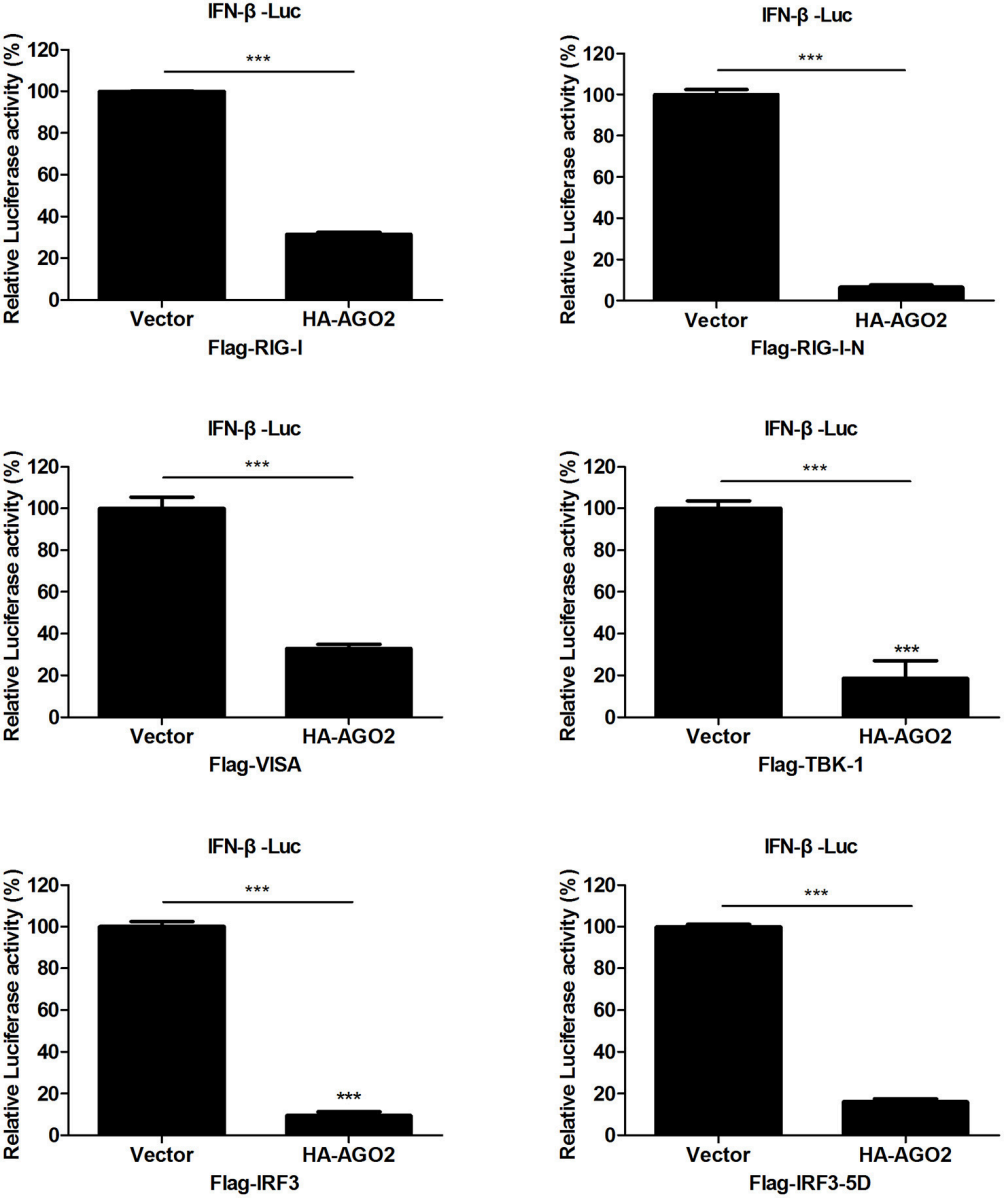
**AGO2 operates at or downstream of IRF3**. HEK293T cells1 were transfected with IFN-β-Luc, pGL4.75 hRluc/CMV, HA-AGO2, or control plasmid, and Flag-RIG-I, Flag-RIG-I-N, Flag-VISA, Flag-TBK-1, Flag-IRF3, Flag-IRF3-5D, or control vectors. After 24 h, cells were collected, and relative luciferase activities were measured. Data are represented as mean ± SD of three experiments. ^***^P < 0.0001, as determined by a *t* test.

Viral infection also leads to activation of RIG-I and TBK-1/IKKε signaling pathway (Katze et al., 2008; Goubau et al., 2013; Wu and Chen, 2014). HEK293T cells were transfected with an expression construct encoding AGO2 and overexpressing each of the signaling molecules RIG-I, RIG-I-N, VISA, TBK-1, IRF3, or IRF3-5D, along with a luciferase reporter plasmid containing the IFN-β promoter (IFN-β-Luc) and pGL4.75 hRluc/CMV to determine the targets of AGO2 inhibition in IRF3 activation signaling cascade. Results showed that AGO2 suppressed activation of the IFN-β promoter; this suppression was mediated by overexpression of RIG-I, RIG-I-N, VISA, TBK-1, and IRF3 (Figure 4). AGO2 also inhibited IRF3-5D induced activation of the IFN-β promoter (Figure 4). These results confirmed that AGO2 negatively regulates activation of IFN-β signaling at the level or downstream of IRF3.

### AGO2 does not affect stability nor inhibit activity of IRF3

**Figure 5:**
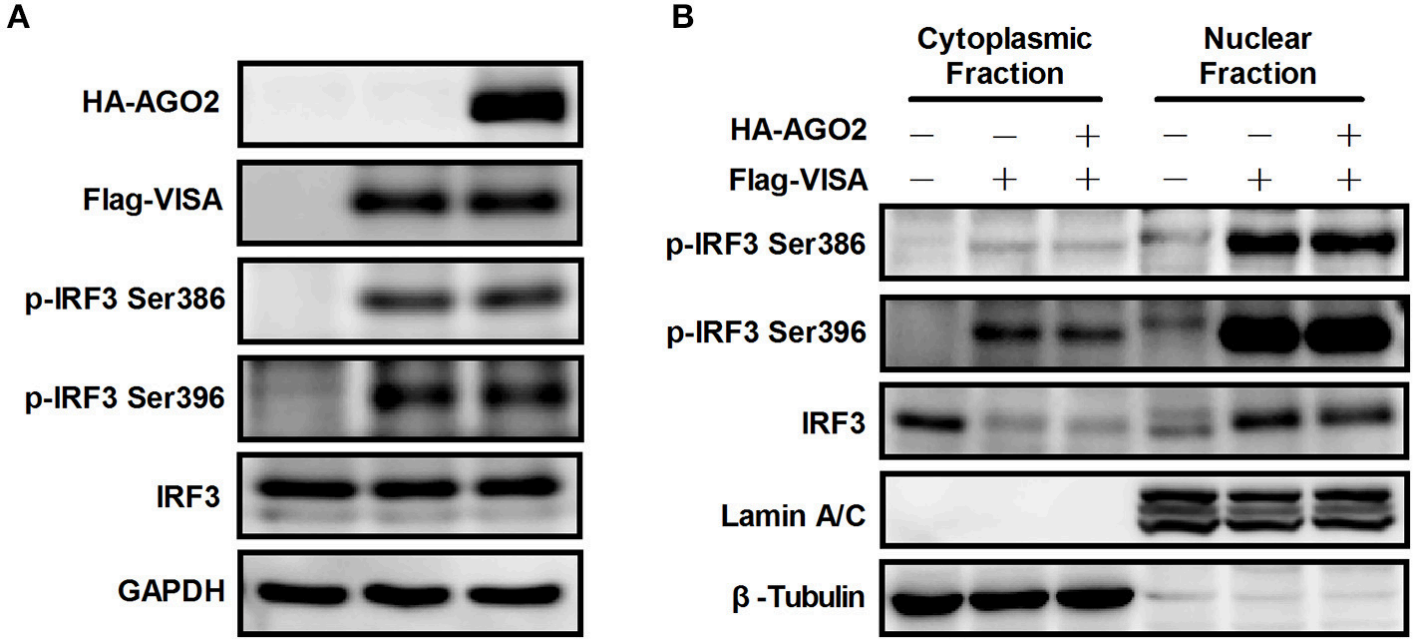
**AGO2 does not affect activation of IRF3. (A)** HEK293T cells were transfected with Flag-VISA and HA-AGO2 with empty vectors as control. After 36 h, cells were lysed, and total IRF3 and Ser386/Ser396 phosphorylated forms of IRF3 were detected by western blot. **(B)** HEK293T cells were transfected with Flag-VISA and HA-AGO2 or empty vectors. After 36 h, subcellular fractionation was implemented to separate the cytoplasm and nucleus from cells. Western blots were performed to detect distribution of total IRF3 and Ser386/Ser396 phosphorylated IRF3 in cells.

IRF3 is phosphorylated and activated by active TBK-1/IKKε upon viral infection (Yoneyama et al., 1998; Chiang et al., 2014). Phosphorylated IRF3 subsequently forms dimers and translocates to the nucleus, where it interacts with transcription coactivators and promotes IFN-β transcription (Chiang et al., 2014; Ysebrant de Lendonck et al., 2014; Song et al., 2016). Ser339 mainly affects protein stability, whereas Ser386/Ser396 play important roles in IRF3 activity (Yoneyama et al., 1998; Servant et al., 2003; Saitoh et al., 2006; Clement et al., 2008). Flag-VISA and HA-AGO2 or an empty vector was overexpressed, and total IRF3 and Ser386/Ser396-phosphorylated IRF3 in HEK293T cells were detected to determine whether the interaction between IRF3 and AGO2 affects stabilization and activation of IRF3. Figure 5A shows that VISA increased phosphorylation of IRF3, but AGO2 did not reduce the protein level of IRF3 nor affected IRF3 phosphorylation.

Although AGO2 did not suppress phosphorylation of IRF3, nuclear translocation following dimerization is also required for proper function of IRF3 (Yoneyama et al., 1998; Lin et al., 1999; Ysebrant de Lendonck et al., 2014). Flag-VISA and HA-AGO2 or an empty vector was overexpressed in HEK293T cells, cytoplasm and nucleus were separated from cells, and western blots were performed to detect the distribution of total IRF3 and Ser386/Ser396 phosphorylated IRF3 in cells. The data showed that AGO2 did not affect nuclear translocation of IRF3 (Figure 5B). Collectively, these results indicated that AGO2 did not decrease protein level (or stability), Ser386/Ser396 phosphorylation, dimerization, and nuclear translocation of IRF3 but possibly inhibited some downstream events after activation of IRF3.

### AGO2 does not affect DNA binding function of IRF3

**Figure 6:**
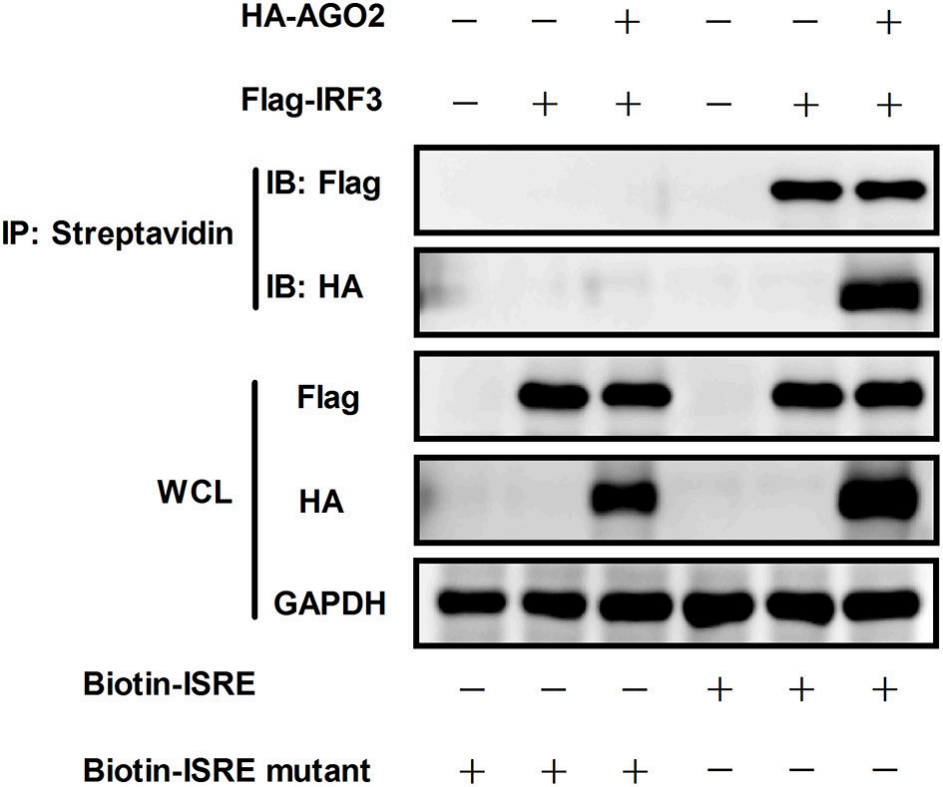
**AGO2 does not influence DNA binding ability of IRF3**. HEK293T cells were transfected with Flag-IRF3 in the absence or presence of HA-AGO2, and cells were infected with SeV at 24 h post transfection. After 12 h, cells were lysed, and cell lysates were mixed with a biotinylated DNA probe (biotin-3 × ISRE or biotin-3 × ISRE mutant) and incubated for 1 h at 4 °C. Then, streptavidin beads were added and incubated overnight at 4°C. The beads were washed four times with PBS and resolved by 2 × SDS loading buffer for western blot.

According to previous studies, IRF3 performs its function by binding to DNA, particularly to IFN stimulated response element (ISRE) sequence, which promotes transcription of IFN-β gene (Weaver et al., 1998). DNA-binding assay was performed to investigate whether AGO2 can prevent this role of IRF3. HEK293T cells were initially transfected with plasmids expressing IRF3 in the absence or presence of AGO2 expression plasmids and were then infected with SeV at 24 h post transfection. Cell lysates were incubated after 12 h with either a native ISRE oligonucleotide, which can interact with activated IRF3 linked to streptavidin beads, or a mutated ISRE oligonucleotide, which cannot interact with activated IRF3 linked to the same beads. The beads were collected, and western blots were performed to detect bound IRF3 in ISRE oligonucleotides. Results indicated that AGO2 did not reduce the binding efficiency between IRF3 and ISRE (Figure 6).

### AGO2 interacts with IRF3

**Figure 7:**
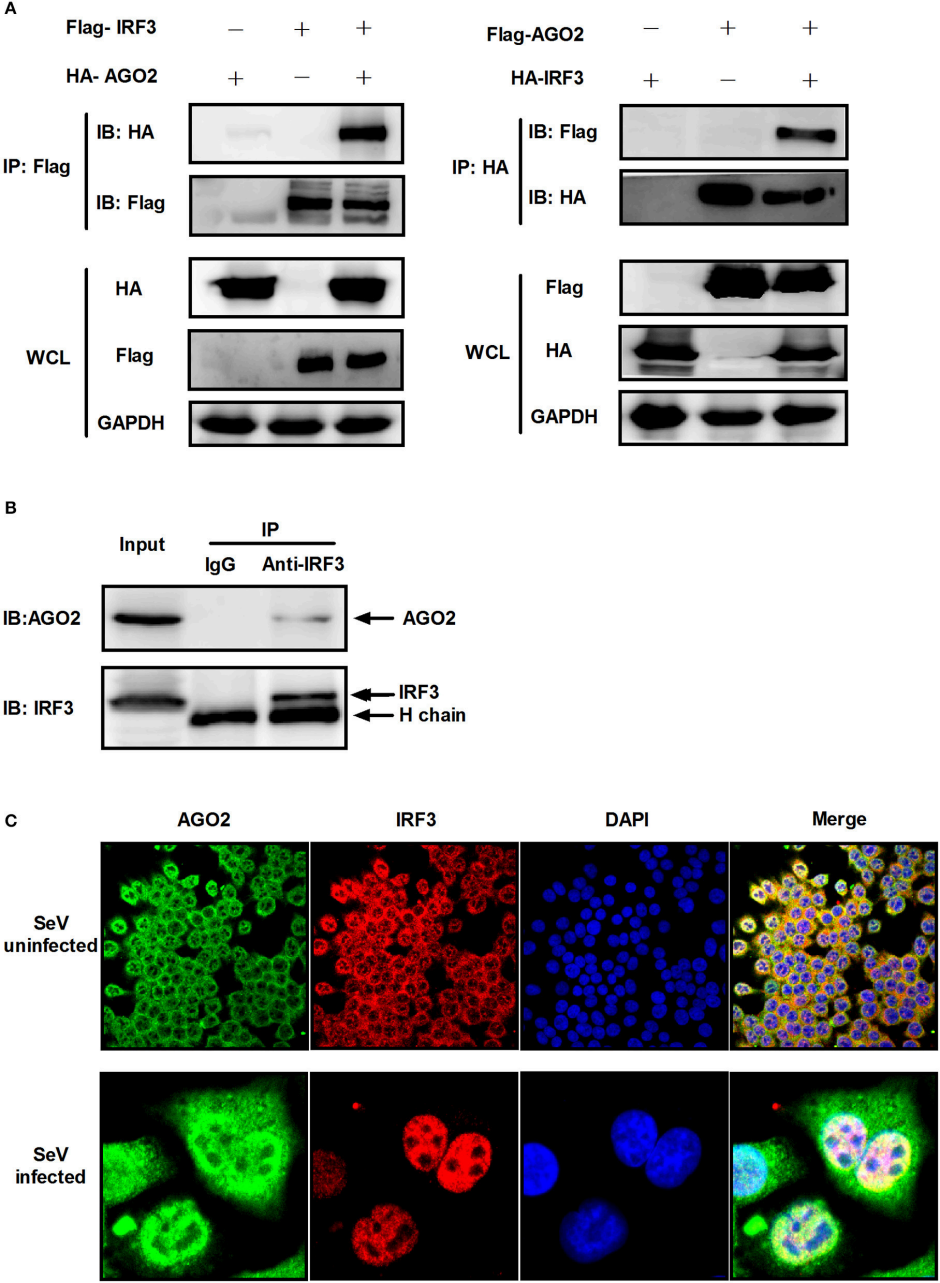
**AGO2 interacts with IRF3 in HEK293T cells. (A)** HEK293T cells were transfected with HA-AGO2 and Flag-IRF3 or with Flag-AGO2 and HA-IRF3. After 36 h, cells were lysed, and immunoprecipitation was performed. **(B)** HEK293T cells were stimulated with SeV for 3 h, and cell proteins were extracted. Anti-IRF3 antibody was used for immunoprecipitation. **(C)** HEK293 cells were mock infected or infected with SeV. At 24 h, subcellular localizations of IRF3 and AGO2 were visualized by immunofluorescence and microscopy.

**Figure 8:**
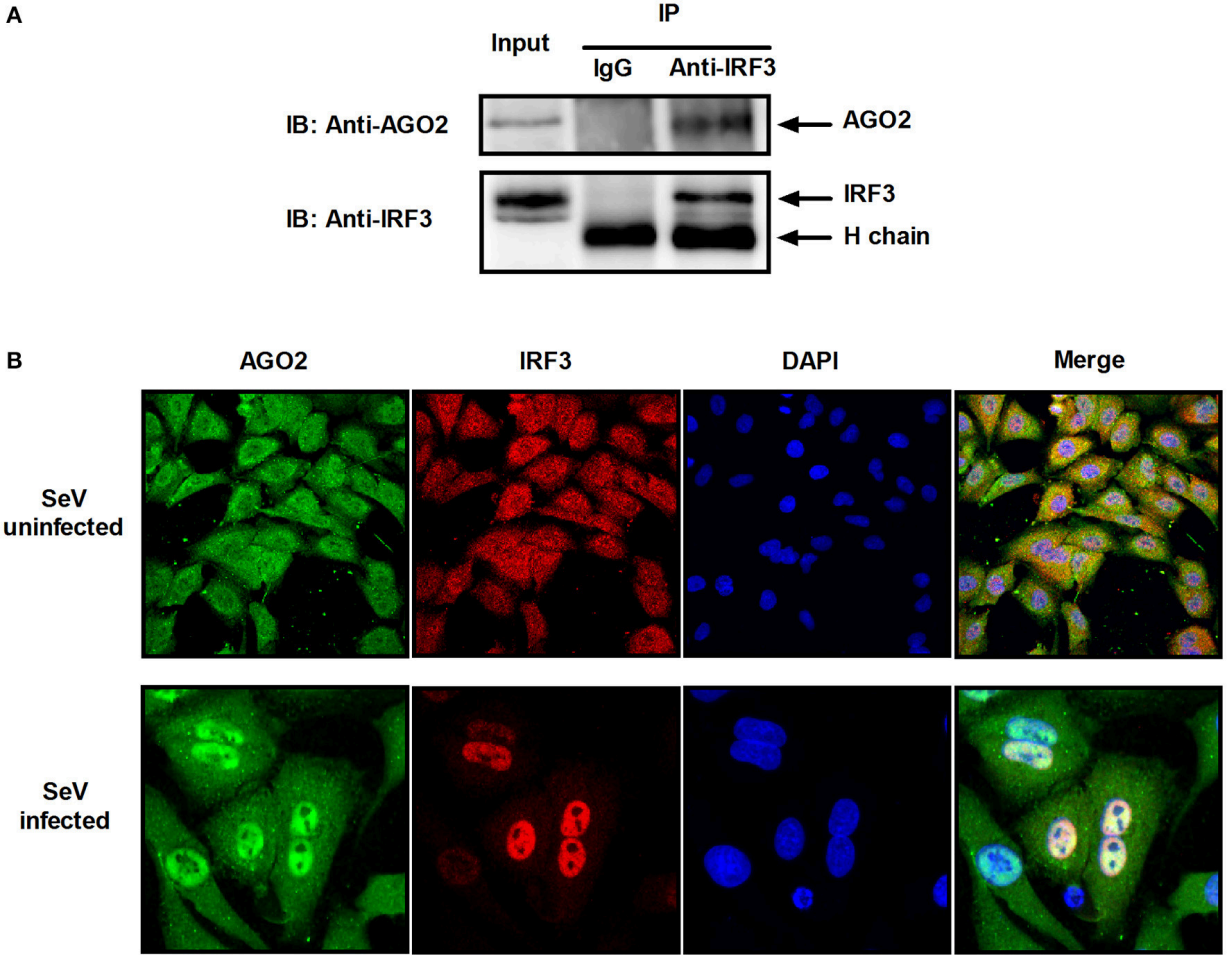
**AGO2 interacts with IRF3 in A549 cells. (A)** A549 cells were stimulated with SeV for 6 h, and cell proteins were extracted. Anti-IRF3 antibody was used for immunoprecipitation. **(B)** A549 cells were mock infected or infected with SeV. At 6 h, subcellular localizations of IRF3 and AGO2 were visualized by immunofluorescence and microscopy.

Previous studies showed that PACT and TRBP can interfere with IFN-β signaling by interacting with signaling molecule RIG-I or PKR (Cosentino et al., 1995; Kok et al., 2011). Immunoprecipitation was performed with HEK293T cells cotransfected with HA-AGO2 and Flag-IRF3 or with Flag-AGO2 and HA-IRF3 to clarify the interaction between AGO2 and IRF3. Both experiments showed that AGO2 interacted with IRF3 (Figure 7A). HEK293T cells were stimulated with SeV for 3 h, proteins were extracted, and anti-IRF3 antibody was used for immunoprecipitation. An endogenous interaction was detected afterward (Figure 7B). Immunofluorescence was also performed in HEK293T cells. IRF3 and AGO2 were co-localized under SeV infected and uninfected cell conditions and were strongly confocal at SeV infected cells (Figure 7C). Endogenous interaction and immunofluorescence was also performed in A549 cells (Figures 8A,B). Both results indicated that IRF3 interacted with AGO2.

### AGO2 interferes with the interaction between IRF3 and CBP/p300 by interacting with C terminus domain of IRF3

**Figure 9:**
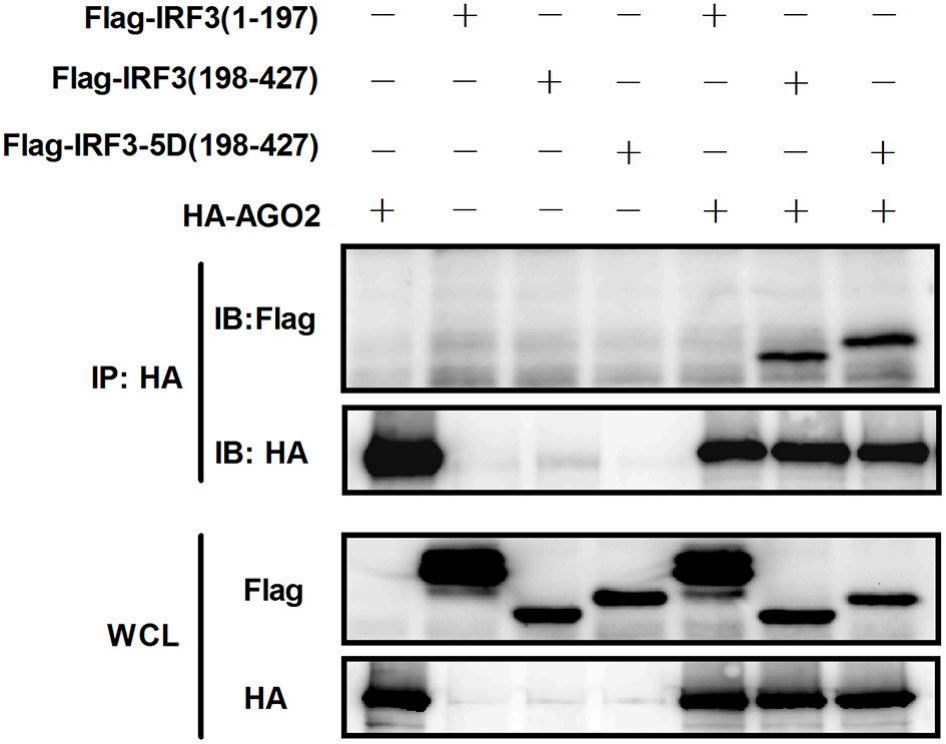
**AGO2 interacts with the C terminal region of IRF3 and IRF3-5D**. HEK293T cells were cotransfected with HA-AGO2 and Flag-IRF3(1–197), Flag-IRF3(198–427), or Flag-IRF3-5D(198–427) with empty plasmid as control. After 36 h, cell proteins were extracted, and immunoprecipitation was performed with anti-HA and anti-Flag antibodies.

**Figure 10:**
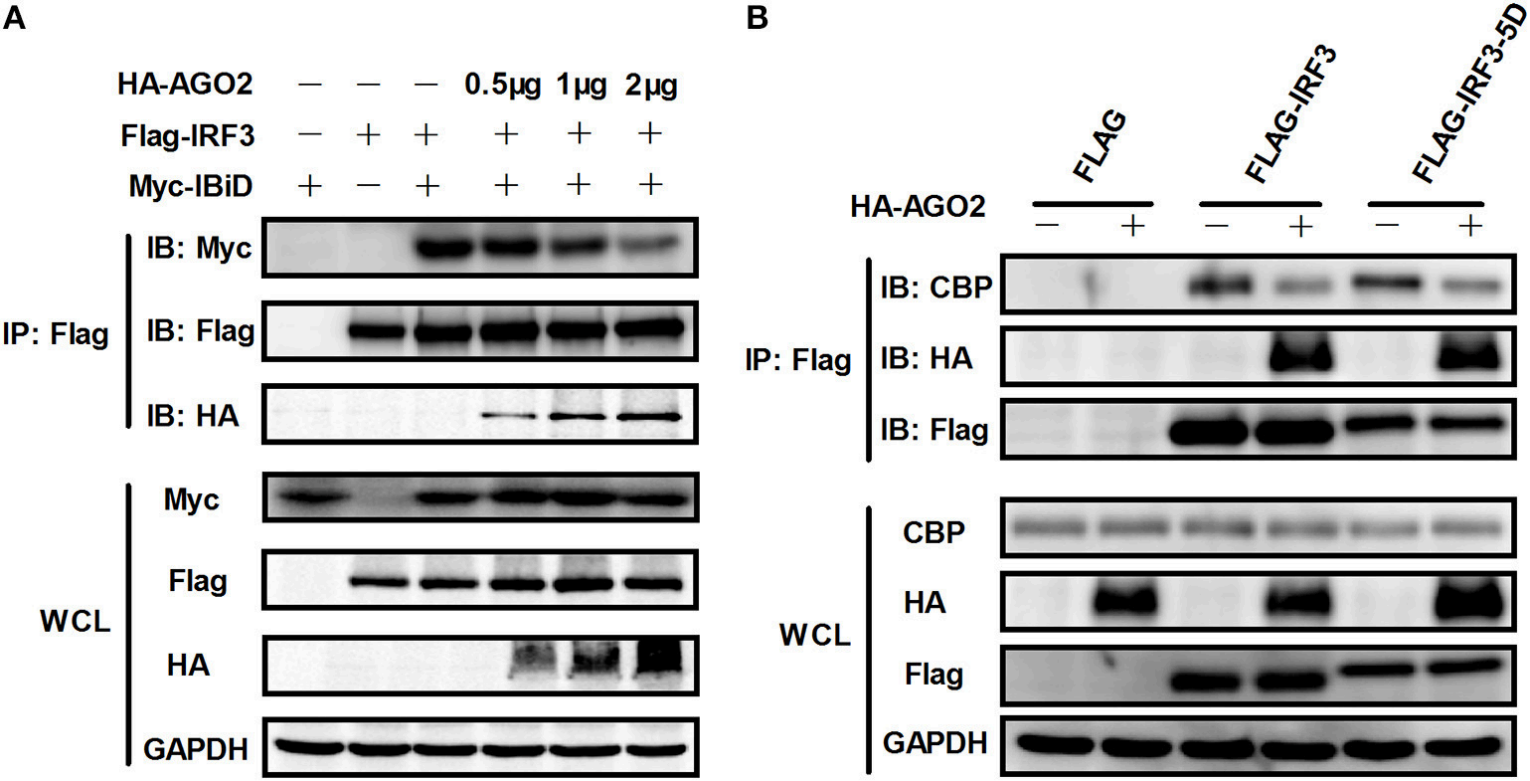
**AGO2 abolishes interaction between IRF3 and CBP/p300. (A)** HEK293T cells were cotransfected with Myc-IBiD, Flag-IRF3, and different concentrations of HA-AGO2 (i.e., 0.5, 1, and 2 μg) with empty plasmid as control. After 36 h, cells were stimulated by SeV for 12 h, and cell proteins were extracted, and immunoprecipitation was performed with anti-HA, anti-Flag, and anti-Myc antibodies. **(B)** HEK293T cells were cotransfected with HA-AGO2 and Flag-IRF3 or Flag-IRF3-5D with empty vectors as control. After 36 h, cells were stimulated by SeV for 12 h, and cell lysates were immunoprecipitated with an anti-Flag antibody and immunoblotted with an anti-CBP antibody.

Given that AGO2 did not alter IRF3 phosphorylation, nuclear translocation, and DNA binding ability, AGO2 was hypothesized to play a role during assembly of IRF3 and CBP/p300 for the formation of transcriptional complex in the nucleus. Two vectors, namely, Flag-IRF3(1–197) containing DNA binding domain (DBD) and Flag-IRF3(198–427)/ Flag-IRF3-5D(198–427) containing the IRF-3 activation domain (IAD), were constructed to examine the role of AGO2 in association of IRF3 with CBP (Lin et al., 1999). Then, each vector was cotransfected with HA-AGO2 in HEK293T cells, and immunoprecipitation was performed. Results showed that AGO2 interacted with the IAD of IRF3/ IRF3-5D, which contains a region that interacted with IBiD (Lin et al., 1999, 2001), implying that AGO2 inhibited the interaction between IRF3 and CBP/p300 (Figure 9). A Myc-IBiD expression vector was constructed and cotransfected with Flag-IRF3 in the absence or presence of different concentrations of HA-AGO2 in HEK293T cells to investigate this hypothesis. Then, immunoprecipitation was conducted, and results showed that AGO2 caused does-dependent inhibition of the interaction between IRF3 and IBiD (Figure 10A). Interaction of CBP with IRF3 and IRF3-5D with cotransfected HA-AGO2 in HEK293T cells was examined to verify further this phenomenon (Figure 10B). All results indicated that HA-AGO2 disturbed the interaction between IRF3 and CBP/p300.

### AGO2 interacts with IRF3 by its MID domain

**Figure 11:**
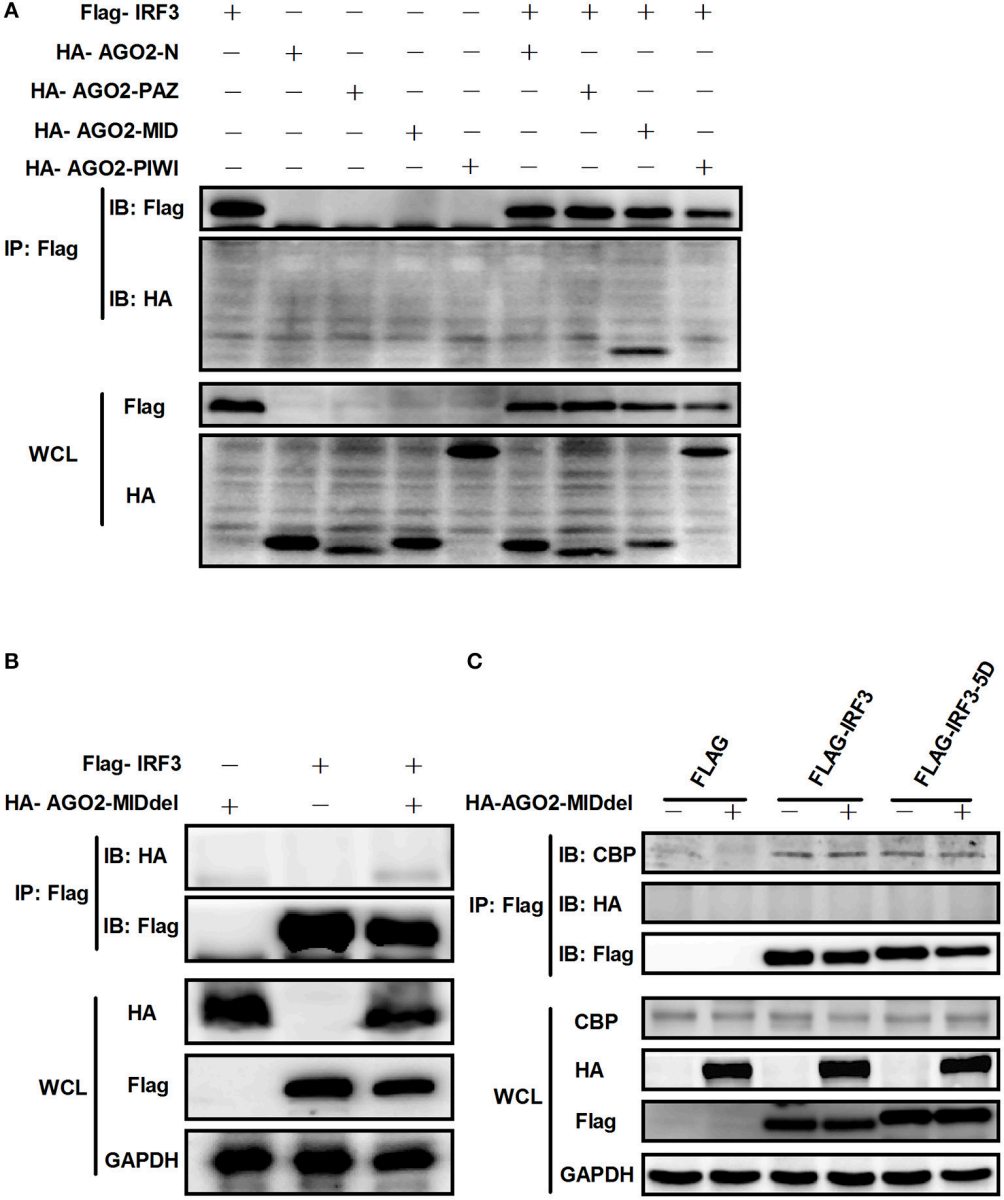
**AGO2 interacts with IRF3 by its MID domain. (A)** HEK293T cells were co-transfected with four truncations of AGO2 and IRF3 and immunoprecipitation was performed at 36 h later. **(B)** The MID deleted mutation of AGO2 (HA-AGO2-MIDdel) and IRF3 were co-transfected into HEK293T cell. After 36 h, immunoprecipitation was performed. **(C)** HEK293T cells were co-transfected with HA–AGO2-MIDdel and Flag-IRF3 or Flag-IRF3-5D with empty vectors as control. After 36 h, cells were stimulated by SeV for 12 h and cell lysates were immunoprecipitated with an anti-Flag antibody and immunoblotted with an anti-CBP antibody.

AGO2 contains four domains which are N domain, PAZ domain, MID domain and PIWI domain (Kuhn and Joshua-Tor, 2013; Ye et al., 2015). We cloned this four domains into pCAGGS-HA expression vector. Then, each expression vector was cotransfected with Flag-IRF3 in HEK293T cells and immunoprecipitation was performed. Results showed that MID domain of AGO2 interact with IRF3 (Figure 11A). A MID domain deleted expression plasmid (HA-AGO2-MIDdel) was constructed and the interaction with IRF3 was examined. Result exhibited that HA-AGO2-MIDdel did not interact with IRF3 (Figure 11B) implying that HA-AGO2-MIDdel could not hinder the interaction between IRF3 and CBP. Interaction of CBP with IRF3 and IRF3-5D with cotransfected HA-AGO2-MIDdel in HEK293T cells was examined to verify this assumption (Figure 11C). All results indicated that AGO2 negatively regulates type I interferon signaling pathway via competition binding IRF3 with CBP/p300.

## Discussion

Also known as eIF2C2, AGO2 belongs to the argonaute family, which participates in RNA-mediated gene silencing (RNAi) pathway (Ye et al., 2015). AGO2 also regulates multiple biological functions through protein–protein, protein–RNA, or protein–DNA interactions (Martinez and Gregory, 2013; Meister, 2013; Ye et al., 2015). Together with AGO2, SETDB1 plays an essential role in transcriptional gene silencing through recruitment of chromatin remodelers and/or other modifiers, consequently creating a repressive chromatin milieu at targeted promoters (Cho et al., 2014). AGO2 can directly bind to the promoter of focal adhesion kinase, which is a critical molecule associated with tumor metastasis, and can trigger its transcription (Cheng et al., 2013). AGO2 also acts as an RNA slicer independent of dicer and as regulator of miRNA maturation (Cifuentes et al., 2010; Ye et al., 2015). Previous studies showed that AGO2 can enhance miRNA stability (Winter and Diederichs, 2011; Ye et al., 2015). The functions of AGO2 remain to be understood. Initial discoveries indicated that this protein may participate in viral replication and type I IFN production in cells.

This study, investigated the influence of AGO2 on virus proliferation. Results showed that knockdown of AGO2 expression inhibited H5N1 virus replication, whereas its overexpression resulted in the opposite phenomenon (Figures 1B,C,E,F). This study also determined that viral infection can modify AGO2 distribution between cytoplasm and cell nucleus, and that it may influence virus/host interactions (Figures 2A,B). Therefore, AGO2 can directly influence innate immune responses and type I IFN signaling pathway. AGO2 was silenced, and expression level of ISGs and IFN-β were detected to investigate this hypothesis. Results indicated that silencing of AGO2 promoted SeV induced expression levels of endogenous IFN-β, downstream IFIT1, and ISGs including Mx1, STAT1, and ISG15 (Figure 3A). The overexpression experiment involving A549 cells and double fluorescence reporting system in HEK293T cells also yielded the same results (Figures 3B–D). The targets of AGO2 inhibition in type I IFN signaling pathway were explored using double fluorescence reporting system in HEK293T cells. Notably, AGO2 suppressed activation of IFN-β promoter, this action was mediated by overexpressions of RIG-I, RIG-I-N, VISA, TBK-1, IRF3, and IRF3-5D (Figure 4). These results indicated that AGO2 targeted at or downstream of IRF3 and negatively regulated activation of IFN-β signaling.

Previous studies illustrated that activation of IRF3 is a key step for activation of IFN-β signaling; IRF3 activation consists of phosphorylation, dimerization, and translocation to the nucleus (Servant et al., 2002; Chen et al., 2008; Takahasi et al., 2010; Ysebrant de Lendonck et al., 2014). Only phosphorylated IRF3 can dimerize and translocate to the nucleus (Lin et al., 1999). The most critical steps comprise phosphorylation and nuclear translocation. Thus, the effects of AGO2 on phosphorylation and nuclear translocation of IRF3 were analyzed using Western blot. Results proved that AGO2 did not prevent phosphorylation and nuclear translocation of IRF3 (Figures 5A,B). Ser339 also affects stability of IRF3 (Saitoh et al., 2006; Clement et al., 2008). Total IRF3 was also detected by western blot, and results showed that total IRF3 did not affected stability of IRF3 (Figure 5A). Binding of IRF3 to ISRE is also an important procedure, and DNA binding assay was performed (Ysebrant de Lendonck et al., 2014; Meng et al., 2016). Results indicated that AGO2 did not interfere with DNA binding ability of IRF3 (Figure 6). All results illustrated that AGO2 did not influence activation and DNA binding ability of IRF3.

Transcriptional coactivators associate with promoters and enhancers primarily through protein–protein contact, and mediate interactions between DNA-bound transcription factors and the general transcription machinery (Torchia et al., 1998; Gusterson et al., 2002; Tsuda et al., 2003). Transcriptional coactivators CBP and p300 are highly homologous and are critical regulators of metazoan gene expression, and interaction between IRF3 and CBP/p300 play a significant role in transcriptional complex formation (Yoneyama et al., 1998; Suhara et al., 2002). CBP/p300 associates with many different DNA-bound transcription factors through small, conserved domains. A previous study identified a compactly folded 46-residue domain (IBiD) in CBP/p300 that can interact with IRF3 at C terminal. IBiD is required for viral induction of IFN-β, and IBiD mutation causes loss of structural integrity of CBP/p300 (Lin et al., 2001).

As AGO2 did not affect activation and DNA binding ability of IRF3 (Figure 6), interfering the formation of transcriptional complex which includes the interaction between IRF3 and CBP/p300, bear significance (Yoneyama et al., 1998; Lin et al., 2001). Immunoprecipitation and immunofluorescence were conducted to understand the mechanisms underlying how AGO2 negatively regulates activation of IFN-β signaling target at or downstream of IRF3. Results showed that AGO2 can interact with IRF3 (Figures 7A–C, 8A,B). Then, Flag-IRF3(1–197), which contains a DBD, and Flag-IRF3(198–427), which contains an IAD domain, were constructed. Each domain was cotransfected with HA-AGO2 in HEK293T cells, and immunoprecipitation was performed. The results indicated that AGO2 interacted with the IRF3 IAD while interacting with the domain associated with IBiD; AGO2 was speculated to interact with IRF3 to reduce IBiD–IRF3 binding (Figure 9). Based on this assumption, Myc-IBiD was constructed and transfected with IRF3 in the absence or presence of HA-AGO2 expressing plasmid. Interaction between IRF3 and IBiD was detected by immunoprecipitation. The data showed that IBiD was dislodged from IRF3 as AGO2 was increased (Figure 10A). Interaction between IRF3/IRF3-5D and endogenous CBP was also detected in the absence or presence of HA-AGO2 expressing plasmid, further proving that AGO2 interfered with the interaction between IRF3 and CBP/p300 (Figure 10B). Therefore, AGO2 sequestered the interaction between IRF3 and CBP/p300. AGO2 contains four domain which is N domain, PAZ domain, MID domain, and PIWI domain (Kuhn and Joshua-Tor, 2013). We funded that MID domain interact with IRF3 and the deletion of MID domain could not abrogate interaction between IRF3 and CBP (Figures 11A,B). The competition binding essay also showed that AGO2-MIDdel could not hinder CBP/p300 binding to IRF3 (Figure 11C). This study provides insight into mechanisms underlying antagonism of IFN by AGO2.

**Figure 12:**
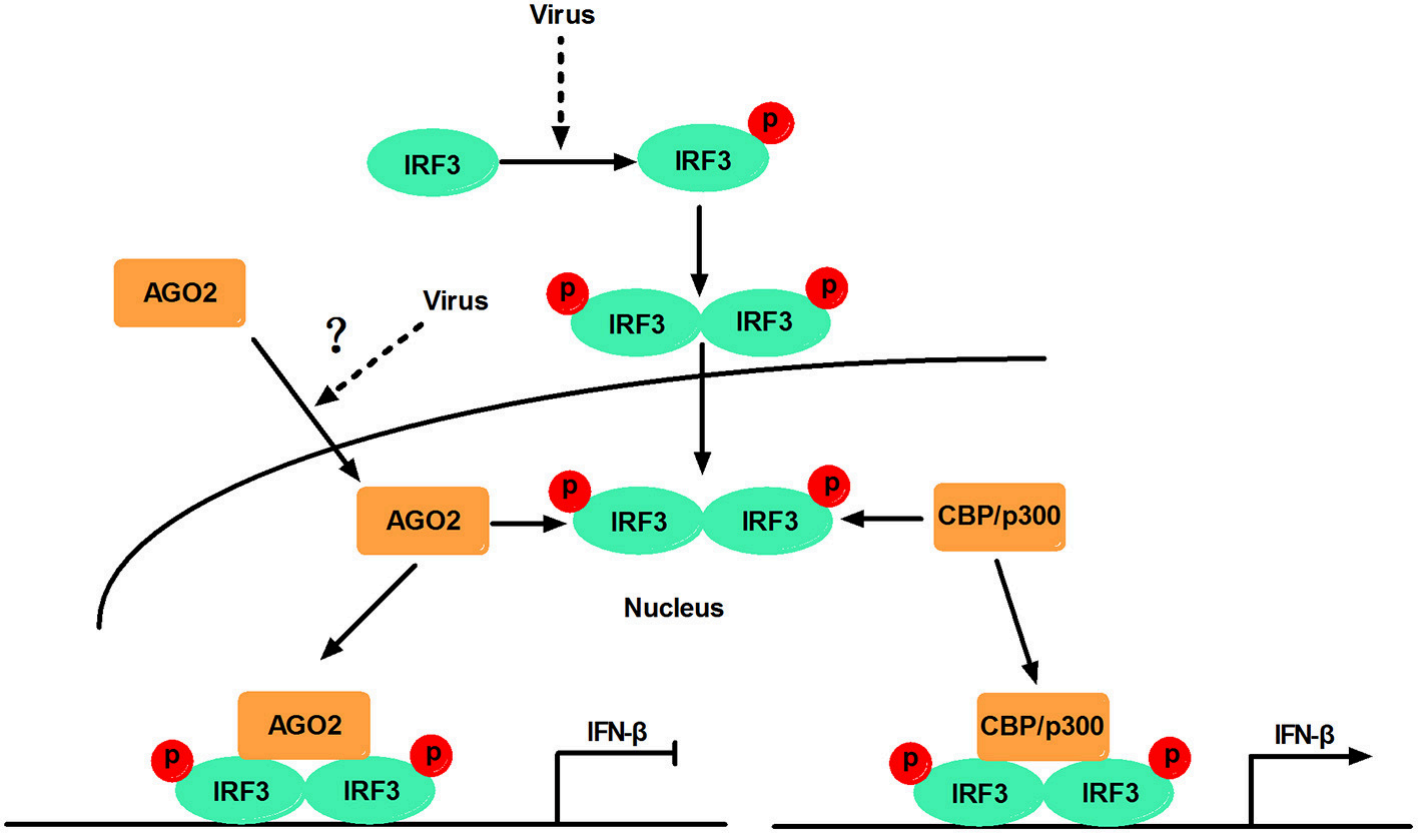
**Schematic diagram of AGO2 action on innate immune signaling pathway**. Viral infection recruits kinases TBK-1 and IKKε to adaptor protein VISA and TRIF. These kinases phosphorylate IRF3, and phosphorylated IRF3 forms dimers that translocate into the nucleus and are activated. Activated IRF3 interacts with CBP/p300, which forms the transcription initiation complex, and induces production of IFN-β. However, AGO2 inhibits IFN-β promoter activation by interfering with IRF3–CBP interaction and represses formation of transcription initiation complex. H5N1 infection can reduce the distribution of AGO2 in the nucleus and further enhance IFN-β promoter activation.

This study, proposed a model in which virus-inducible activation of IRF3 interacts with coactivator CBP/p300 and permits IRF3 primary activation of IFN-β and IFN-β responsive genes. This study also showed that AGO2 serves as a negative regulator, which suppresses IFN-β and IFN-β responsive genes by disturbing IRF3 binding to CBP/p300, and that the interaction between IRF3 and AGO2 poses no influence on IRF3 DNA binding capacity. H5N1 can induce IFN-β expression by decreasing nuclear AGO2 protein, which can inhibit IFN-β expression in the nucleus (Figure 12).

AGO2 is a potential novel factor that maintains balance of in virus-induced type I IFN signaling pathway. This study also proposed a theory that cells can downregulate AGO2 content in the nucleus when stimulated by viruses, to induce inhibitory effects of AGO2 on IFN-β expression and upregulate expression of IFN-β. Then, viruses can take advantage of inhibitory mechanisms of AGO2 to further propagate in cells. Previous studies showed that AGO2 and RNAi factors Dicer, TRBP, and TRNC6A/GW182 are located in the nucleus and associate together in multiprotein complexes (Gagnon et al., 2014). IPM8 is a regulator of AGO2, and knockdown of IPM8 reduces the nuclear AGO2 pool (Weinmann et al., 2009). The subcellular distribution of AGO2 during H5N1 infection depends on RNAi factors (Dicer, TRBP, and TRNC6A/GW182), IPM8 or other mechanisms, which should be further investigated. The proposed theory come and the observed phenomena will benefit prevention of virus infection.

## Author contributions

MJ, HC, and SW conceived and designed the experiments; SW, XinS, CY, and DZ performed the experiments. SW and MJ analyzed the data; XinS, CY, XL, and XiaS contributed reagents/materials/analysis tools; SW wrote the paper. All authors have read and approved the final draft.

## Funding

This work was supported by National Key Research and Development Program of China (No. 2016YFD0500205) and National Key Research and Development Program of China (No. 2017YFD0501600).

### Conflict of interest statement

The authors declare that the research was conducted in the absence of any commercial or financial relationships that could be construed as a potential conflict of interest.
